# Apparent mineralocorticoid excess: comprehensive overview of molecular genetics

**DOI:** 10.1186/s12967-022-03698-9

**Published:** 2022-11-03

**Authors:** Yi-ting Lu, Di Zhang, Qiong-yu Zhang, Ze-ming Zhou, Kun-qi Yang, Xian-liang Zhou, Fan Peng

**Affiliations:** Department of Cardiology, Fuwai Hospital, National Center for Cardiovascular Diseases, Chinese Academy of Medical Sciences and Peking Union Medical College, Beijing, China

**Keywords:** Apparent mineralocorticoid excess, *HSD11B2* gene, 11β-HSD2, Genetic testing, Hypertension, Non-classic

## Abstract

Apparent mineralocorticoid excess is an autosomal recessive form of monogenic disease characterized by juvenile resistant low-renin hypertension, marked hypokalemic alkalosis, low aldosterone levels, and high ratios of cortisol to cortisone metabolites. It is caused by defects in the *HSD11B2* gene, encoding the enzyme 11β-hydroxysteroid dehydrogenase type 2 (11β-HSD2), which is primarily involved in the peripheral conversion of cortisol to cortisone. To date, over 50 deleterious *HSD11B2* mutations have been identified worldwide. Multiple molecular mechanisms function in the lowering of 11β-HSD2 activity, including damaging protein stability, lowered affinity for the substrate and cofactor, and disrupting the dimer interface. Genetic polymorphism, environmental factors as well as epigenetic modifications may also offer an implicit explanation for the molecular pathogenesis of AME. A precise diagnosis depends on genetic testing, which allows for early and specific management to avoid the morbidity and mortality from target organ damage. In this review, we provide insights into the molecular genetics of classic and non-classic apparent mineralocorticoid excess and aim to offer a comprehensive overview of this monogenic disease.

## Introduction

Apparent mineralocorticoid excess (AME, OMIM: 218030) is a rare form of monogenic hypertension that is transmitted as an autosomal recessive trait. The clinical symptoms of AME were first reported in 1974 by Werder et al. in a 3-year-old girl with low birth weight, delayed growth, polydipsia, polyuria, and hypertension. In 1977, New et al. identified patients with similar symptoms, characterized their biochemical profiles, and named the disease AME [1, 2]. Initially, it was speculated that *HSD11B1* (encoding 11β-hydroxysteroid dehydrogenase type 1 [11β-HSD1]) was the causative gene but no mutation was detected in AME patients; thus, the focus was shifted to other candidate genes [3]. In 1995, Wilson et al. identified the first *HSD11B2* mutation in several siblings with typical characteristics of AME from a consanguineous Iranian family, unraveling the genetic defects of AME [4]. The molecular pathogenesis of AME primarily results from a deficiency in the enzyme 11β-hydroxysteroid dehydrogenase type 2 (11β-HSD2), which is involved in the peripheral metabolism of cortisol [5, 6]. In 1999, Nunez et al. summarized the AME genotype–phenotype correlation by studying 14 affected children and proposed that clinical and/or biochemical parameters and enzyme activity were closely related [7].

A timely diagnosis of AME is pivotal because continuous poor management of blood pressure and potassium can cause end organ damage such as early stroke, hypertensive myocardial hypertrophy, hypertensive retinopathy, and deterioration of renal function [8]. Genetic analysis, regarded as a unique method for the accurate diagnosis of disease, continually broadens the genetic spectrum of AME [9]. In recent decades, great progress has been made in understanding the pathogenesis of AME, which aids the development of targeted therapy [10] and a novel clinical condition with a mild phenotypic spectrum named non-classic AME was identified. Examining *HSD11B2*-related genetic or non-genetic determinants has important implications in understanding the special condition. This review summarizes the clinical presentation, pathophysiology, molecular genetic basis, and genetic testing of AME.

## Pathophysiology

**Fig. 1:**
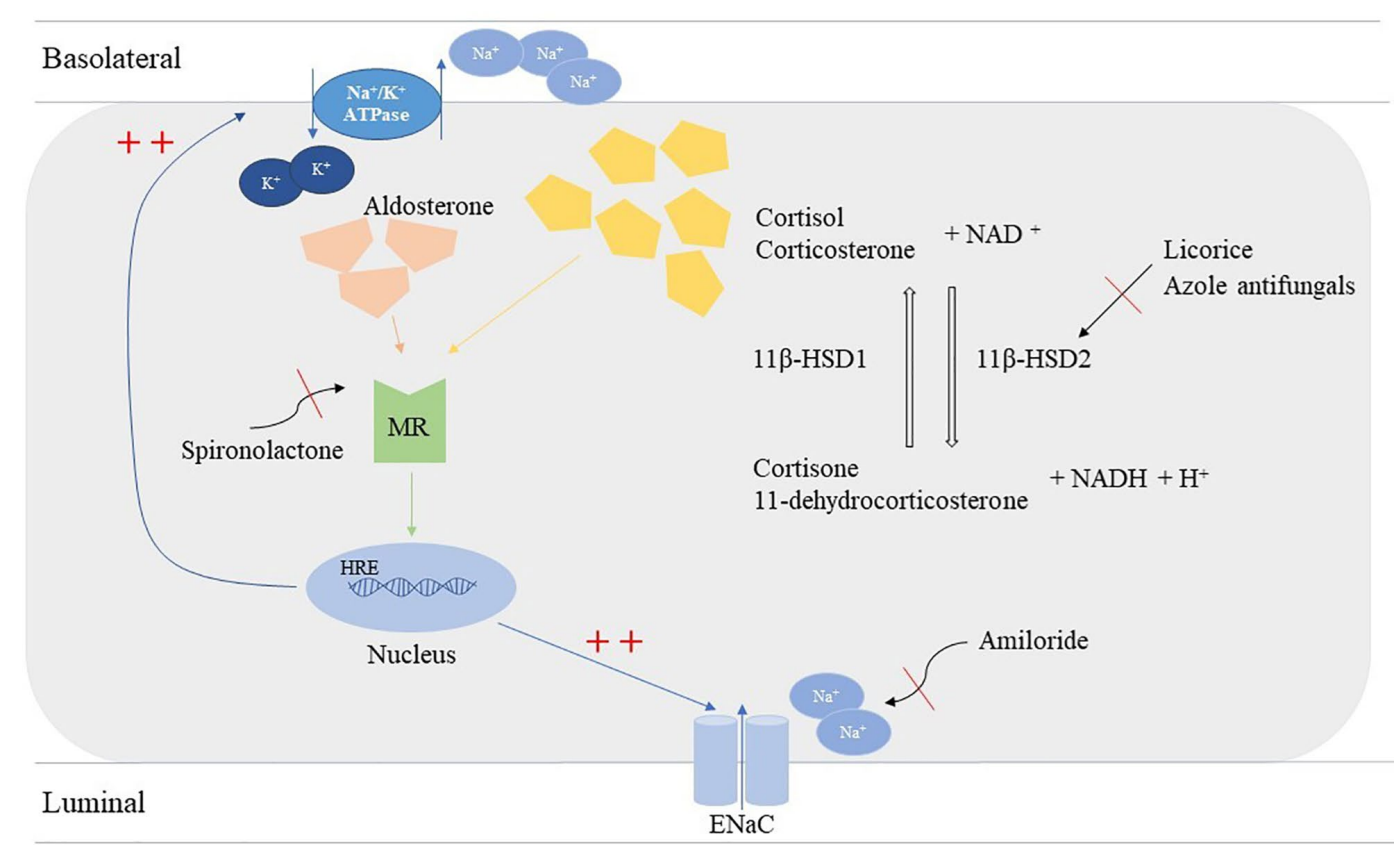
Mechanisms of apparent mineralocorticoid excess syndrome. 11β-HSD2 enzyme facilitates the conversion from active cortisol to inactive metabolite form, cortisone; 11β-HSD1 has an opposing function. Physically, aldosterone and cortisol are both ligands of mineralocorticoid receptors, which results in binding to nuclear hormone response elements, leading to transcription of Na^+^/K^+^ ATPase and EnaC channels. The deficiency of 11β-HSD2 fails in the metabolism of cortisol and results in excessive mineralocorticoid, so as the over-ingestion of licorice and azole antifungals causes the same effects. Mineralocorticoid receptor blocker, spironolactone, could block the hyperactivation of mineralocorticoid receptors while amiloride blocks EnaC remarkably.

Cortisol is a hormone secreted by the zona fasciculata of the adrenal cortex, which plays a crucial role in cognition, development, metabolism, the immune system, and the stress response [11, 12]. Cortisol levels are regulated by two isoforms of 11β-HSD: 11β-HSD1 and 11β-HSD2. 11β-HSD2 is widely distributed in various tissues, such as the brain, placenta, kidney, and colon [13], and facilitates the conversion of active steroid cortisol to its inactive metabolite form, cortisone; 11β-HSD1 has the opposing function (Fig. 1). Both cortisol and aldosterone are ligands of mineralocorticoid receptors (MRs). In vitro, MRs have equal affinity for both cortisol and aldosterone while in vivo MRs are much stronger for aldosterone than cortisol [14]. The role of 11β-HSD2 in cortisol metabolism is to mediate the ligand selectivity of aldosterone for MRs, and the full abolishment or partial activity loss of 11β-HSD2 leads to the continuous accumulation of cortisol and MR overstimulation following the upregulation of sodium reabsorption, increased potassium loss, and low-level renin [5, 15]. In addition, following the failure of the conversion of cortisol, the excretion of urinary cortisol metabolites tetrahydrocortisol (THF) and allo-THF increases and the cortisone metabolite tetrahydrocortisone (THE) decreases [16]. Moreover, it is worthy to note that 11β-HSD2 distributes more widely in the fetus than after birth [17]. Feto-placental 11β-HSD2 is regarded as a ‘glucocorticoid barrier’ which ensures most maternal cortisol inactivated strictly determining the fetal homeostasis of cortisol [18]. Abolished 11β-HSD2 in the placenta causes the fetus to be over-exposed to maternal glucocorticoids, leading to the phenotype of intrauterine growth restriction associated to glucocorticoid receptor [19, 20].

Additionally, excessive ingestion of exogenous 11β-HSD2 inhibitors such as licorice and azole antifungals results in MR induced hypertension; thus, taking a detailed personal and pharmacological history is beneficial when identifying acquired forms of AME [21–23].

## Phenotype of AME

AME is a rare disorder and the prevalence of AME across the hypertensive population has yet to remain unclear. Consistent with the nature of autosomal recessive inheritance, predominant causative mutations occur in consanguineous or endogamous groups or in families affected by a founder effect (Table 1) [8, 24–27]. Moreover, no sex predominance is distributed for the disease [28]. Depending on its phenotypic severity, AME can be divided into two forms: classic AME and non-classic AME (Table 2) [29].

**Table 1:** Clinical manifestations, biochemical profile of AME patients identified by genetic analysis

Patient number	Family number	Ethnicity	Consanguinity	Mutation	Gender	LBW	Clinical manifestations						Biochemical profile						Reference
							Early-onset HT	GR	Hx of FTT	Hx of polyu/polyd	Nephrocalcinosis	Other complications	Serum K^+^, mmol/L	PAC/PRA/PRC	MA	u THFs/THE	u F/E	Others	
1	1	Iranian	Y	R337C	F	Y	Y	Y	Y	N	N	hypertensive nephropathy	↓	PAC↓, PRA↑	-	↑	-	-	[4, 8, 28]
2	1	Iranian	Y	R337C	F	Y	Y	Y	Y	Y	N	-	↓	↓	-	↑	-	-	[4, 8, 28]
3	1	Iranian	Y	R337C	M	Y	Y	Y	Y	Y	N	-	↓	↓	-	↑	-	-	[4, 8, 28]
4	2	Indian	Y	R337C	M	-	Y	-	-	-	Y	LVH, Hx of hypokalemic paralysis	↓	↓	-	-	-	albuminuria	[76]
5	2	Indian	Y	R337C	M	-	Y	-	-	-	Y	LVH, Hx of hypokalemic paralysis	↓	↓	-	-	-	-	[76]
6	3	Native North American	-	R208C	M	-	Y	-	-	-	-	-	-	-	-	↑	-	-	[5]
7	4	Omani	N	R208C	M	Y	Y	Y	Y	-	Y	facial palsy	↓	↓	Y	↑	-	-	[28, 78]
8	4	Omani	N	R208C	M	Y	Y	Y	Y	-	Y	facial palsy	↓	↓	Y	↑	-	-	[28, 78]
9	5	Saudi Arabian	Y	R208C	F	Y	Y	Y	Y	Y	Y	mild LVH	↓	↓	Y	-	↑	hypercalciuria	[79]
10	5	Saudi Arabian	Y	R208C	F	Y	Y	Y	Y	-	Y	-	↓	↓	Y	-	↑	hypercalciuria	[79]
11	5	Saudi Arabian	Y	R208C	M	Y	Y	Y	Y	-	Y	-	↓	↓	Y	-	↑	hypercalciuria	[79]
12	6	-	Y	R208H	F	Y	Y	Y	Y	Y	Y	LVH, hypertensive retinopathy	↓	↓	Y	-	-	-	[80]
13	7	Native South American/Caucasian	-	R213C	F	-	Y	Y	Y	Y	-	retinal vasoconstriction	↓	↓	-	↑	-	-	[5, 81]
14	7	Native South American/Caucasian	-	R213C	F	-	Y	Y	Y	Y	-	-	↓	-	-	↑	-	-	[5, 81]
15	8	Algerian	Y	R213C	M	N	Y	-	-	Y	Y	moderate LVH	↓	↓	Y	↑	-	hypercalciuria	[39]
16	9	French	N	R213C	F	Y	Y	-	-	-	Y	renal failure	↓	↓	Y	↑	-	-	[39]
17	10	Chilean	Y	R213C	F	Y	Y	-	-	-	Y	LVH	N	↓	-	↑	↑	-	[35]
18	11	Native North American/Caucasian	-	L250P, L251S	F	-	Y	-	-	-	-	-	-	-	-	↑	-	-	[5]
19	12	Native North American	-	L250P, L251S	M	-	Y	-	-	-	-	-	-	-	-	↑	-	-	[5]
20	13	Native North American/Caucasian	-	L250P, L251S	M	-	Y	-	-	Y	-	pyloric stenosis	↓	↓	Y	↑	-	-	[5, 81]
21	14	Native American (Chippewa)	Y	L250P, L251S	M	-	Y	Y	-	-	Y	-	↓	↓	-	↑	-	-	[78]
22	15	East Indian	N	R337_Y338delinsH	M	Y	Y	Y	Y	Y	Y	LVH, hypertensive reto	↓	↓	Y	↑	-	-	[5, 82, 83]
23	16	Iranian	N	R337_Y338delinsH	M	Y	Y	-	-	-	N	-	↓	↓	-	↑	-	-	[28, 78]
24	17	Northern Indian	N	R337_Y338delinsH	M	-	Y	N	-	Y	N	-	↓	↓	-	↑	-	-	[78]
25	18	Northern Indian	N	R337_Y338delinsH	M	Y	Y	-	-	-	Y	-	↓	↓	Y	↑	-	-	[28, 78]
26	19	Mexican/American	-	c.664 + 14 C > T	F	-	HT	-	-	-	-	-	-	-	-	↑	-	-	[5]
27	20	Irish/American	-	*Y232_T234del* G305Afs*48	M	-	Y	-	-	-	-	-	-	-	-	↑	-	-	[5]
28	21	African American	N	R186C	F	Y	Y	N	-	-	Y	hypertensive retinopathy, LVH	↓	↓	Y	↑	↑	-	[28, 78, 82]
29	21	African American	N	R186C	F	N	Y	N	-	-	Y	LVH, cerebral palsy, deafness	↓	↓	Y	↑	↑	-	[28, 78, 82]
30	22	Brazilian	Y	R186C	M	Y	Y	Y	Y	Y	Y	mild LVH	↓	N	Y	-	-	hypercalciuria	[84]
31	23	Native American	N	E356Vfs*40	F	Y	Y	Y	Y	N	N	died of LVH	↓	↓	Y	↑	-	-	[2, 78]
32	24	Asian	Y	R374*	M	-	Y	Y	Y	Y	Y	LVH, DI	↓	↓	Y	↑	↑	hypercalciuria	[20, 85]
33	24	Asian	Y	R374*	M	-	Y	Y	-	Y	Y	-	↓	-	-	↑	-	hypercalciuria	[20, 85]
34	24	Asian	Y	R374*	M	-	-	-	-	-	-	stillbirth at 28 weeks’ gestation	-	-	-	-	-	-	[20, 85]
35	24	Asian	Y	R374*	M	-	-	-	-	-	-	stillbirth at 28 weeks’ gestation	-	-	-	-	-	-	[20, 85]
36	25	Iranian	Y	R374*	M	-	Nor	-	Y	-	Y	-	↓	-	Y	-	-	-	[74]
37	26	North European	N	c.1218 + 10 C > T	F	Y	Y	-	-	-	-	dilated aorta descendens, arachnodactylia, scoliosis, lens subluxation, stroke (19 y), Marfan syndrome	↓	↓	Y	↑	-	-	[86]
38	26	North European	N	c.1218 + 10 C > T	M	Y	Y	Y	Y	Y	-	retinopathy, subluxation of the ocular lenses, LVH, hydronephrosis	↓	↓	Y	↑	-	-	[86, 87]
39	27	Brazilian	Y	A328V	F	-	Y	Y	Y	Y	-	nocturia, retinopathy, LVH	↓	↓	Y	↑	-	-	[88, 89]
40	28	Portuguese	Y	A328V	M	N	Y	-	-	-	-	LVH, renal cortical atrophy	↓	↓	N	↑	-	-	[39]
41	28	Portuguese	Y	A328V	M	N	Y	Y	-	-	-	LVH	↓	↓	N	↑	-	proteinuria, hypercalciuria	[39]
42	29	Japanese	N	*R208H* *R337_Y338delinsH*	M	N	Y	-	-	Y	-	retinopathy, LVH	↓	↓	Y	↑	-	-	[49, 90]
43	30	Italian/Moroccan	N	*L250R* *D244N*	M	Y	Y	N	Y	Y	Y	LVH, I-grade hypertensive retinopathy	↓	↓	Y	↑	-	hypercalciuria	[8]
44	31	Turkish	N	L287Cfs*36	M	N	Y	N	Y	Y	Y	congenital left ptosis	↓	↓	Y	↑	↑	-	[8, 28]
45	32	Italian	Y	R279C	F	-	HT	-	-	-	-	-	↓	-	-	↑	-	-	[91]
46	32	Italian	Y	R279C	M	-	Y	Y	-	Y	Y	-	↓	-	-	↑	-	-	[91]
47	32	Italian	Y	R279C	F	-	Y	Y	-	Y	Y	-	↓	-	-	↑	-	-	[91]
48	32	Italian	Y	R279C	F	-	HT	-	-	-	-	-	↓	-	-	↑	-	-	[91]
49	33	North American (Mennonite)	Y	P227L	F	N	Y	N	N	N	N	-	N	↓	N	↑	-	-	[28, 32]
50	34	Japanese	-	S180F	F	Y	Y	-	-	-	-	DI, paralysis	↓	-	-	↑	-	-	[7]
51	35	Caucasian/Australian	-	*L179R* *F246 + 1nt*	M	Y	Y	-	Y	-	Y	DI, LVH	↓	-	-	↑	↑	-	[7]
52	36	Caucasian	-	*A237V* *A328V*	M	N	Y	-	-	-	-	-	↓	-	-	↑	↑	-	[7]
53	37	Caucasian	N	*A237V* *A328V*	M	N	Y	-	-	Y	Y	mild LVH, renal cysts	↓	↓	Y	↑	-	-	[33]
54	38	Mexican-American	-	*R208H* *c.664 + 14 C > T*	F	N	Y	-	Y	-	-	DI	↓	-	-	↑	↑	-	[7]
55	39	-	Y	L114_E115del	F	-	Y	Y	-	-	Y	LVH	↓	↓	Y	↑	↑	hypercalciuria	[55]
56	39	-	Y	L114_E115del	M	-	Y	Y	-	-	Y	LVH	↓	↓	Y	↑	↑	hypercalciuria	[55]
57	40	Omani	Y	L114_E115del	M	Y	Y	-	Y	Y	Y	cardiac arrest (3.5 y)	↓	-	Y	↑	-	-	[9]
58	41	Caucasian	-	*Y226N* *c.1393 C > T* *V254V*	M	-	HT	-	-	Y	Y	nocturia, cardiac arrest (21 y), III-grade retinopathy, renal cyst, LVH	↓	↓	-	↑	-	-	[38, 92]
59	42	Italian	N	*R359W* *c.664 + 1G > A*	F	-	Y	-	-	-	-	cerebral aneurysm	↓	↓	-	↑	↑	-	[38]
60	43	Caucasian	-	*Y232C* *L376P*	M	-	Y	-	-	-	-	type 1 diabetes, hypertensive renal damage	↓	↓	-	↑	-	-	[38]
61	44	Chilean	-	D223N c.664 + 14 C > T	M	-	Y	Y	Y	Y	Y	-	↓	↓	-	-	serum ↑	-	[40]
62	45	Kuwaiti	Y	A273V	F	Y	Y	Y	-	Y	Y	-	↓	↓	Y	↑	-	-	[44]
63	45	Kuwaiti	Y	A273V	F	N	Y	Y	-	-	Y	-	↓	-	Y	↑	-	-	[44]
64	46	-	-	A273V	F	Y	Y	-	Y	-	-	DI	↓	-	Y	-	-	-	[93]
65	47	Omani	Y	R74G P75Rfs*42	M	N	Y	-	Y	Y	Y	-	↓	-	Y	↑	-	-	[9]
66	47	Omani	Y	R74G P75Rfs*42	M	Y	Y	-	-	-	-	mild LVH, congestive heart failre (0.5y)	↓	-	Y	-	-	-	[9]
67	48	Omani	-	R74G P75Rfs*42	M	Y	Y	-	-	Y	N	mild LVH	↓	-	Y	↑	-	-	[9]
68	49	Omani	Y	A221V	M	Y	Y	-	Y	Y	N	mild LVH, respiratory failure (4.2 y)	↓	-	Y	↑	-	-	[9]
69	49	Omani	Y	A221V	F	Y	Y	-	Y	Y	N	-	↓	-	Y	↑	-	-	[9]
70	49	Omani	Y	A221V	F	Y	Y	-	-	-	Y	-	N	-	N	↑	-	-	[9]
71	49	Omani	Y	A221V	F	Y	Y	-	Y	Y	N	-	↓	-	-	↑	-	-	[9]
72	50	Omani	-	V321_V322insAPV	M	Y	Y	-	Y	Y	-	mild LVH	↓	-	Y	↑	-	-	[9]
73	50	Omani	-	V321_V322insAPV	F	Y	Y	-	-	-	-	mild LVH	↓	-	-	-	-	-	[9]
74	51	Pakistani	Y	Y299del	F	Y	Y	-	-	-	Y	dilation of the aortic root, retinopathy	↓	↓	Y	↑	-	-	[50]
75	52	French	N	*D144V* *F367del*	M	N	Y	Y	-	-	N	LVH, tetanic convulsions	↓	↓	N	↑	-	-	[39]
76	53	French	-	F185S	F	Y	Y	-	-	-	N	LVH	N	↓	N	↑	-	-	[39]
77	54	-	-	F185S	F	-	Y	-	Y	Y	Y	Coffin-Siris syndrome, dilatation in the aortic root	↓	↓	Y	-	-	-	[94]
78	55	Moroccan	Y	P381Pfs*22	M	N	Y	N	-	-	Y	mild LVH	↓	↓	Y	↑	-	-	[39]
79	56	-	-	*Y338H* S26*	F	Y	Y	Y	Y	Y	-	end-stage renal failure, subarachnoidal hemorrhage	↓	↓	-	Plasma ↑	Plasma ↑	-	[1, 52]
80	57	-	Y	D176N	M	N	Y	N	-	-	N	-	↓	↓	Y	-	-	-	[95]
81	58	-	Y	A221G	M	N	Y	N	-	-	N	mild LVH	↓	↓	-	↑	-	-	[25]
82	58	-	Y	A221G	M	N	Y	N	-	-	-	-	↓	↓	-	↑	-	-	[25]
83	59	Qatari	Y	G89D	F	Y	Y	Y	Y	-	-	LVH, cyst fibrosis	↓	↓	Y	-	-	-	[24]
84	59	Qatari	Y	G89D	M	Y	Y	Y	-	-	-	distal ileal obstruction, LVH, cyst fibrosis	↓	↓	Y	-	-	-	[24]
85	60	Omani	Y	T267A	M	Y	Y	-	Y	Y	Y	aortic root dilation	↓	↓	N	-	-	-	[56]
86	60	Omani	Y	T267A	F	N	Y	-	-	-	Y	aortic root dilation, LVH	↓	↓	Y	↑	-	-	[56]
87	60	Omani	Y	T267A	F	Y	Y	-	-	-	Y	-	↓	↓	N	-	-	-	[56]
88	60	Omani	Y	T267A	F	N	Y	-	-	-	N	LVH, renal calculi, aortic root dilation	↓	↓	Y	↑	-	-	[56]
89	60	Omani	Y	T267A	F	N	Y	-	-	-	Y	aortic root dilation, LVH	↓	↓	N	↑	-	-	[56]
90	60	Omani	Y	T267A	F	Y	Y	-	-	-	Y	-	↓	↓	N	↑	-	-	[56]
91	61	Chinese	N	L363P	M	Y	Y	Y	-	Y	-	basal ganglion hemorrhage	↓	↓	-	-	↑	-	[54]
92	62	Syrian	Y	G296_I300del	M	Y	Y	Y	Y	-	Y	cholelithiasis	↓	↓	Y	-	↑	-	[96]
93	62	Syrian	Y	G296_I300del	F	Y	Y	N	-	-	-	borderline LVH	↓	-	Y	-	-	-	[96]
94	63	Pakistani	Y	E301Rfs*56	F	Y	Y	Y	Y	Y	Y	mild LVH	↓	↓	Y	↑	-	hypercalciuria	[97]
95	64	Chinese	N	*E115_L116del* *F367del*	M	-	Y	-	-	-	Y	enlargement of the left atrium and ventricle, mild mitral regurgitation, renal cyst	↓	↓	Y	-	-	proteinuria	[77]
96	65	-		F367I	F	Y	Y	Y	-	Y	Y	renal medullary cysts, LVH	↓	-	N	-	-	-	[98]
97	-	Omani	Y	E115_L116del	F	Y	Y	-	-	-	Y	-	↓	↓	Y	↑	-	-	[28]
98	-	Omani	Y	R74Gfs*43	F	Y	Y	-	-	-	Y	-	↓	↓	Y	↑	-	-	[28]
99	-	Omani	Y	R74Gfs*43	M	Y	Y	-	-	-	Y	-	↓	↓	N	↑	-	-	[28]
100	-	Omani	Y	R74Gfs*43	F	Y	Y	-	-	-	Y	-	↓	↓	Y	↑	-	-	[28]
101	-	Omani	Y	R74Gfs*43	M	Y	Y	-	-	-	Y	-	↓	↓	Y	-	-	-	[28]
102	-	Omani	Y	R74Gfs*43	M	Y	Y	-	-	-	Y	-	↓	↓	Y	↑	-	-	[28]
103	-	Omani	Y	R74Gfs*43	M	Y	Y	-	-	-	N	-	↓	↓	N	-	-	-	[28]
104	-	Omani	Y	R74Gfs*43	M	Y	Y	-	-	-	Y	-	↓	↓	Y	↑	-	-	[28]
105	-	Omani	Y	R74Gfs*43	M	Y	Y	-	-	-	Y	-	↓	↓	N	↑	-	-	[28]
106	-	Omani	Y	A221V	F	Y	Y	-	-	-	Y	-	↓	PAC ↓, PRA ↑	N	-	-	-	[28]
107	-	Omani	Y	A221V	F	Y	Y	-	-	-	Y	-	↓	↓	N	-	-	-	[28]
108	-	Omani	Y	V321_V322insAPV	M	Y	Y	-	-	-	Y	-	↓	↓	Y	↑	-	-	[28]
109	-	Omani	Y	V321_V322insAPV	F	Y	Y	-	-	-	Y	-	↓	↓	Y	-	-	-	[28]
110	-	Omani	Y	V321_V322insAPV	M	Y	Y	-	-	-	Y	-	↓	↓	Y	↑	-	-	[28]

AME, apparent mineralocorticoid excess; Italic mutations mean compound heterozygous mutations; -, not available; Y, yes; N, normal; M, male; F, female; LVH, left ventricular hypertrophy; PAC, plasma aldosterone concentration; PRC, plasma renin concentration; PRA, plasma renin activity; MA, metabolic alkalosis; LBW, low birth weight (defined as less than 2.5 kg); early-onset hypertension is defined as an increasement in blood pressure aged 18 years or less; Hx of FTT, history of failure to thrive; Hx of polyu/polyd, history of polyuria/polydipsia; GR, growth retardation; DI, nephrogenic diabetes insipidus; u THFs/THE, urinary (tetrahydrocortisol + 5α-tetrahydrocortisol)/ tetrahydrocortisone or urinary (tetrahydrocortisol + allo-tetrahydrocortisol)/ tetrahydrocortisone; u F/E, urinary cortisol to cortisone; the italics represent complex heterozygous mutations

**Table 2:** Indicative symptoms and markers suggested for classic AME and non-classic AME

	Classic AME	Non-classic AME
Phenotypes		
Range of age at diagnosis	Infant to juvenile	Adolescent to adult
Blood pressure	III grade hypertension or higher	Normal /mild hypertension
History of polyuria and polydipsia	Y	N
Pre- and postnatal growth failure	Y	N
Failure to survive	Y	N
Markers		
Electrolyte	Hypokalemia	Normal
Plasma renin activity	Low	Low
Plasma aldosterone level	Low	Normal
Urinary F/E	High	Slightly increased
Urinary THF + allo-THF/THE	High	Normal/slightly increased
Others	Exosomal urinary HSD11B2 mRNA	Microalbuminuria, plasminogen activator inhibitor-1, sensitivity c-reactive protein, L-dopachrome, gamma-L-glutamyl-L-methionine sulfoxide, 5-sulfoxymethylfurfural, S-phenylmercapturic acid, bilirubin, L-iditol, deoxyribose 1-phosphate, citric acid TNF

AME, apparent mineralocorticoid excess; Y, yes; N, normal; F, cortisol; E, cortisone; THF, tetrahydrocortisol; THE, tetrahydrocortisone

### Classic AME

Despite the abolishment of 11β-HDS2 leads to deranged cortisol metabolism and decreased urinary excretion of cortisol metabolites, patients with AME have normal serum cortisol concentration and don’t present with clinical features of Cushing’s syndrome or Addison’s disease [14, 30]. Presumably, the prolonged cortisol half-life may result in the low rate of cortisol secretion because of normal hypothalamic-pituitary-adrenal axis (HPA) regulation effect [14, 31]. Classic AME usually starts in infancy to juvenile and typically manifests as low birth weight, refractory hypertension, delayed growth, polyuria and polydipsia, failure to thrive [32]. Marked hypokalemia, metabolic alkalosis, suppressed plasma renin activity, low levels of aldosterone, and increased urinary or serum cortisol to cortisone ratios are typical characteristics of classic AME. Additionally, nephrocalcinosis and renal cysts are common, possibly ascribing to chronic long-standing hypokalemia [33, 34].

### Non-classic AME

Patients with non-classic AME, also known as AME type 2, present with milder phenotypes including slight hypertension and subtle biochemical disturbances, which is proposed as a novel clinical condition different from classic AME [32]. In the literature, non-classic AME is commonly presented in adolescents or adults which develops much later than classic AME. Mainly, non-classic AME is characterized by a high urinary cortisol/cortisone ratio and low cortisone level [35]. Different from classic AME, blood pressure level of non-classic AME is normal or slightly elevated, approximately 141.0/88.5 mm Hg [36]. Recently, a cross-sectional study identified a partial defect of 11β-HSD2 in 7.1% of a primary care cohort in Chile [37].

These patients are often undetected, and the disease is not usually diagnosed until adulthood. By analyzing metabolic changes of non-classic AME patients and healthy controls, Tapia‑Castillo et al. found gamma-L-glutamyl-L-methionine sulfoxide and 5-sulfoxymethylfurfural might be sensitive biomarker of non-classic AME [36]. Moreover, high levels of inflammatory markers, microalbuminuria, high-sensitivity C-reactive protein, plasminogen activator inhibitor-1 are indicated in non-classic AME [37].

Of note, some heterozygous AME subjects with a single pathogenic mutation display various manifestations, ranging from normal to mild or moderate phenotype [38, 39]. It is either haploinsufficiency or the dominant mutant negative effect that partially explain this phenotypic variability [38, 40].

## Treatment and long-term follow-up

For the nature of AME is a kind of salt-sensitive hypertension, salt-limited diet is necessary for both classic and non-classic AME patients [41, 42]. Targeting the pathogenic pathway, MR antagonist (spironolactone or eplerenone) combined with potassium sparing diuretics are strongly recommended for both AME individuals with satisfactory curative effects. Generally, MR antagonist for classic AME at doses ranging from 2 to 10 mg/kg/day while low dose of MR antagonist (12.5–25 mg/day) is advised for non-classic AME [43, 44]. Glucocorticoids also have been administrated for suppressing the secretion of endogenous adrenocorticotropic hormone-mediated corticosteroid in adult classic AME patients [45]. Moreover, kidney transplantation has also been reported in classic AME patients which was shown to “cure” AME [46, 47]. So far, there is few data regarding the long-term follow-up of classic AME patients. Razzaghy-Azar et al. followed an Iranian family of three sibs affected with classic AME for 20 years and found that the eldest sibling with the longest delay in diagnosis developed left ventricular dysfunction and renal failure who had to undergo renal transplantation while other two sibs didn’t suffer from end-organ damage [47]. In 2017, Yau et al. reported long-term follow-up results of a large series comprising 36 classic AME patients, including cardiovascular mortality (19%), persistence of nephrocalcinosis (89%), and kidney failure (15%) [28]. The long-term outcome associated with non-classic AME patients remains unclear.

## Genetics of AME

### *HDS11B2* gene

**Fig. 2:**
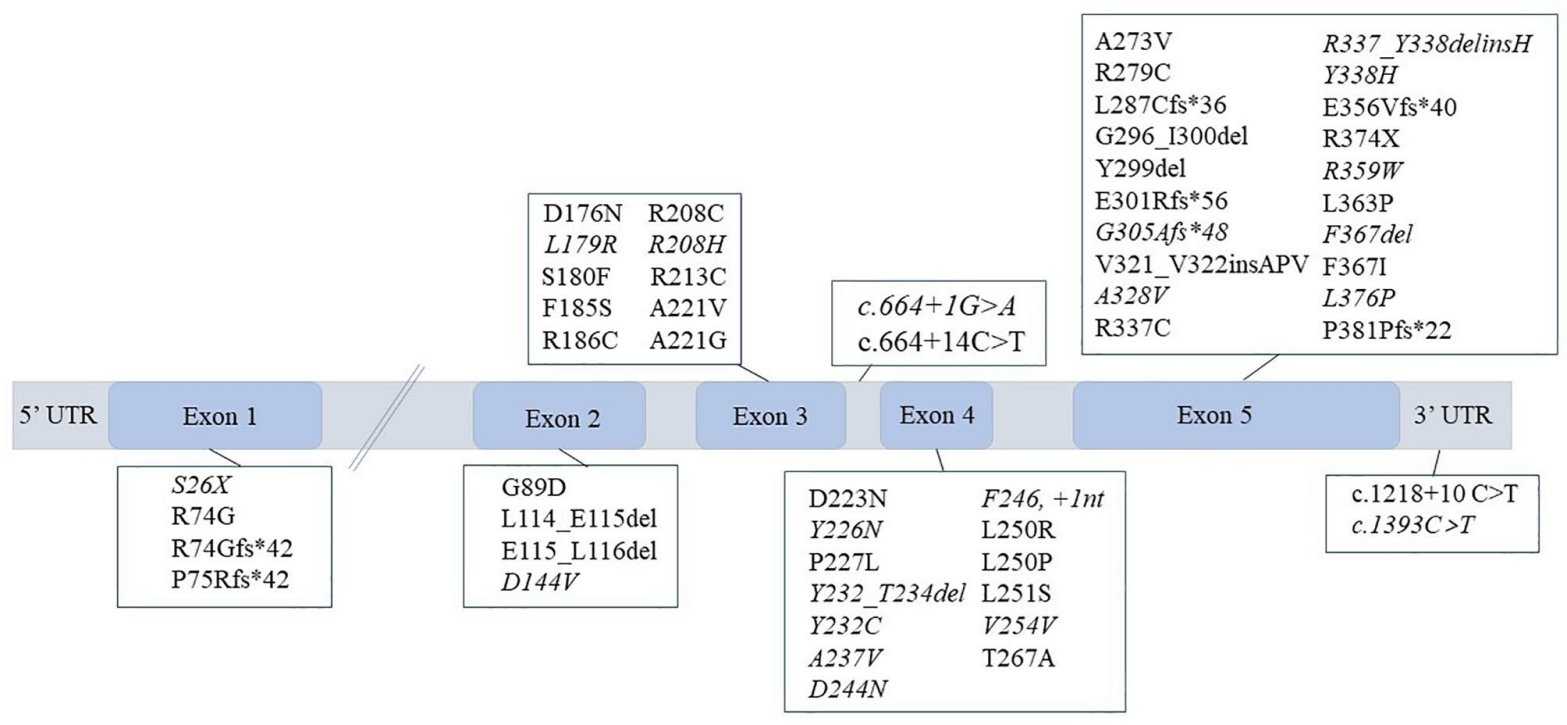
Mutant spectrum of *HSD11B2* gene. The italics represent compound heterozygous mutations, and the rest are homozygous mutations.

The *HSD11B2* gene is located on chromosome 16q22.1, has an approximate length of 6.2 kb, and consists of five exons [48]. Since the first *HSD11B2* mutation (p.Arg337Cys) was identified, a total of 51 deleterious mutations have been reported that are causative of AME (Fig. 2) [4]. Most pathogenic mutations occur in exons 3–5, indicating that these regions are critical for maintaining stable activity of 11β-HSD2 and/or have an active tendency to mutate [49]. Missense mutations at single points are the most frequent mutation type, although nonsense, frameshift, and splicing mutations leading to truncated, inactive proteins, have also been discovered at low rates. Several harmful splicing mutations have been reported that have varying effects on enzyme activity. For example, the nucleotide substitution of C to T in intron 3 (*IVS3 + 14 C > T*) changes the structure of the pre-mRNA, affects normal splicing, and results in the absence of exon 4 which encodes the catalytic domain; thus, the translated 11β-HSD2 protein is inactive [5, 7, 40]. The *IVS3 + 1G > A* mutation affects the splice donor site, causing incomplete expression of exon 3. This allows some normal splicing thus constituting moderate impairment of 11β-HSD2. However, the mutant partially reserves some normal splicing process followed by a moderate impairment in 11β-HSD2 [38]. Although the *de novo* mutation c.771 C > G, identified in a Caucasian family, is silent (p.Val254Val), the mutant minigene contains an aberrant consensus donor splice site that results in a premature truncation in exon 5 [38].

### Molecular pathogenesis of classic AME

11β-HSD2 is a nicotinamide adenine dinucleotide (NAD)-dependent dehydrogenase of 405 amino acids that contains two main domains: the cofactor (NAD^+^) binding region and the substrate binding region. 11β-HSD2 only functions in its catalytic role when the coenzyme binding site binds to NAD^+^ and the substrate binding site binds to cortisol. Previous work established mutant transfectants to investigate enzyme activity and expression in vitro or used an *in silico* model to predict potential impacts on protein function, thereby identifying possible pathogenic mechanisms [40, 50, 51]. Any mutations potentially influencing protein stability, the affinity to the substrate or cofactor, and the dimer interface have been found to impair enzymatic activity and cause classic AME [38, 51–53]. Moreover, genetic polymorphism, environmental factors as well as epigenetic modifications may also offer an implicit explanation for the molecular pathogenesis of non-classic AME by having an increased salt appetite or affecting *HSD11B2* expression.

#### Impaired stability of 11β-HSD2 protein

The loss of 11β-HSD2 protein stability accelerates its degradation rate in a pathway thought to contribute to the development of classic AME. The functional validation of mutant 11β-HSD2 in vitro by Nunez et al. revealed a more significant reduction in the enzymatic activity of mutants Ser180Phe, Ala237Val, and Ala328Val in cell lysates than in whole cells, indicating impaired enzyme stability [7]. The Arg/Tyr amino acid cluster (residues 335–339) has been shown to be of great importance for 11β-HSD2 stability [52]. Several AME cases carrying mutations at this cluster have been reported to have significantly decreased 11β-HSD2 activity [4, 5, 49, 52]. As enzymatic stability decreases, the mutant protein degradation rate increases, and its half-life reduces. Compared with wild-type 11β-HSD2 with a half-life of 21 h, Tyr338His and Arg337Cys mutations reduce the half-life to 3 and 4 h, respectively [52]. Of note, the Tyr338His mutation disrupted the normal endoplasmic reticulum and microsome localization of 11β-HSD2, and the mutant protein was detected in perinuclear bodies which also may influence its stability [52].

#### Attenuated affinity for the substrate

The substrate binding region of 11β-HSD2 is composed of a chain of highly conserved residues, and it is thought that the catalytic activity mechanism may rely on the interaction between specific hydrophobic residues with cortisol. Pizzolo et al. identified the novel homozygous Ala221Gly substitution, which had a severely deleterious effect on 11β-HSD2 activity [25]. As shown in an *in silico* model, the hydrophobic side chain of Ala221 causes it to be closely associated with the substrate binding site. However, the conversion of alanine to the polar hydrophilic amino acid glycine destroys the local hydrophobic environment and attenuates the affinity of 11β-HSD2 for cortisol [25]. Similarly, the Tyr226Asn mutation alters the hydrophobic side chain to a hydrophilic one, which attenuates hydrophobic interactions and disrupts the substrate interaction [28]. Recently, Wang et al. showed that the substrate–protein interaction site might be located in a shallow pocket within residues 357–367 [54]. They also detected a novel missense mutation, Leu363Pro, with decreased affinity for cortisol that appears due to lack of local hydrophobicity [54].

#### Blunted affinity to the cofactor

Specific mutations associated with the coenzyme binding pocket in 11β-HSD2 enzyme weaken its affinity for NAD^+^. In 2001, Odermatt identified a consecutive deletion mutation (Leu114_Glu115del) in *HSD11B2*, which is the first site shown to decrease the affinity of the protein for the coenzyme. They found that the negative charge of glutamic acid at position 115 reduced the binding preference for NAD^+^. Moreover, the loss of amino acids Leu114 and Glu115 led to an abnormal conformation of the coenzyme binding region and disturbed the transformant efficiency of the electron [55]. More recently, Yau et al. observed that the hydroxyl side chain of threonine at site 267 interacts with the amide nitrogen of the coenzyme to form a hydrogen bond, which helps the NAD^+^ localize with coenzyme binding domain. The Thr267Ala missense mutation impairs the hydrogen bond structure, thus affecting the alignment of NAD^+^ in the coenzyme binding pocket [56]. By constructing a model of the Asp223Asn mutant protein structure, Carvajal et al. found that alterations in the electrostatic potential of the enzyme surface contribute to weakening its affinity for NAD^+^ [40].

#### Disruption of the dimer interface

11β-HSD2 functions as a monomer, or a homodimer under inactive conditions [57, 58]. The dimer interface consists of a portion of helix, including vital residues such as Arg186, Ala237, Asp244, and Leu251 and so on [28, 51]. By disrupting inter-subunit ion pair interactions, hydrogen bond interactions, or salt bridge interactions at the interface, mutations can enhance the formulation of homodimers, thus abolishing the activity of 11β-HSD2 [28].

### Other potential mechanisms associated with non-classic AME

Excessive dietary salt intake is a known risk factor and the appetite for salt is susceptible to the activity of 11β-HSD2 in the brain. In the adult brain, the only site where MRs and 11β-HSD2 are co-expressed is inside the nucleus of the solitary tract, while the subcommissural organ and the ventromedial hypothalamic nucleus merely express 11β-HSD2 [59]. Notably, all of these regions are involved in modulating the appetite for salt [59–61]. Previously, Ingram et al. reported a case of AME with an increased salt appetite [62]. Moreover, animal model observed that basal blood pressure of brain-specific *HSD11B2* knockout mice was similar to that of healthy control mice, but they gradually went on to develop hypertension for a three- fold increased salt consumption that could be inhibited by spironolactone [63]. It has been speculated that defective 11β-HSD2 in both central and renal systems simultaneously affects sodium homeostasis and contributes to the phenotype of hypertension [63].

As well as genetic deficiencies, epigenetic modifications also play a crucial role in regulating *HSD11B2* expression in the onset of hypertension with underlying defects in 11β-HSD2 [64]. By analyzing *HSD11B2* expression in vitro and in vivo in rats, Alikhani-Koopaei et al. found that high methylation of CpG islands in the *HSD11B2* promoter reduced gene expression, which was potentially associated with hypertension, and that the inhibition could be reversed by inducing demethylation [65]. A close relationship exists between the extent of *HSD11B2* promoter DNA methylation and adverse birth outcomes, such as low birth weight and insufficient gestational age at delivery, emphasizing the inhibiting effect of placental DNA methylation in fetal intrauterine growth [66, 67]. In addition, it is reported that the download expression of miRNA (miR‑192‑5p and miR‑204‑5p) in urinary exosomes also plays a potential role regulating the phenotype [68]. Santis et al. identified that the expression level of exosomal urinary *HSD11B2* mRNA was closely associated with the hypertension phenotype [69].

In terms of genetic polymorphism, Alikhani-Koupaei et al. found that frequent G209A polymorphism was associated with salt sensitivity that reduced the transcription and expression of *HSD11B2* by hindering the binding of nuclear factor 1 and glucocorticoid receptor to its promoter [70]. Collectively, genetic or epigenetic modifications as well as environmental factors (age, sodium intake) may compose multiple hits, responsible for the molecular genetics of AME phenotypic differences [71–73].

## Genetic testing for AME

If not diagnosed and treated in time, chronic hypertension and hypokalemia alkalosis may cause extensive, severe consequences, including diseases of the renal, central nervous, cardiovascular, and retinal systems, or even sudden fatality [8]. We suggest that patients with clinical symptoms resembling AME should undergo 24-h urinary steroid quantification to determine the profile of cortisol and cortisol metabolites. Traditional laboratory evaluation findings have been shown to be ambiguous in terms of ruling out other monogenic disorders with similar clinical and hormonal patterns, such as Liddle syndrome, Batter syndrome and primary glucocorticoid resistance [10, 74, 75].

Identifying specific causative mutations utilizing genetic testing is a confirmatory tool in the diagnosis of AME. Because 11β-HSD2 can be deficient in low-renin hypertensives, genetic testing of *HSD11B2* is required to screen for AME, especially in patients with a clinical history or increased cortisol to cortisone ratios [32]. Moreover, with an increasing number of asymptomatic cases detected among individuals with a positive family history, it is necessary to conduct genetic counseling in affected families to clarify genetic involvement [56]. Patients with high-risk pathogenic mutations require close monitoring to ensure that the disease is well controlled [8, 38]. Initially, Wilson et al. designed the intron and exon derived primer sequences of the targeted gene for repeat PCR amplification of exons and discovered an identical missense mutation site in two affected siblings [4]. Since then, more causative mutations have been discovered. Promisingly, as next-generation sequencing technology becomes more common, whole exome sequencing has emerged as a cost-effective tool to detect pathogenic mutations, and to be particularly suitable in patients with atypical and overlapping clinical features or for whom biochemical profiles are unavailable [74, 76, 77].

## Conclusion

In summary, AME is an autosomal recessive form of infant or juvenile low-renin hypertension caused by deleterious mutations in *HSD11B2*. Such mutations disrupt the stability of the protein or dimer interface, and cause a loss of affinity for substrate and/or cofactor, thus attenuating or abolishing 11β-HSD2 activity. Because of the heterogeneity of AME clinical manifestations, it is challenging to accurately diagnose the disease early in clinical practice. Genetic testing is pivotal in the precise identification of AME and guides subsequent treatment to prevent end organ damage and sudden death. Considering the potential mechanisms of non-classic AME, genetic polymorphism, environmental factors as well as epigenetic modifications associated with *HSD11B2* should be highlighted.

## Data Availability

Not applicable.

## Abbreviations

**11β-HSD2**: 11β-hydroxysteroid dehydrogenase type 2.
**AME**: Apparent mineralocorticoid excess.
**11β-HSD1**: 11β-hydroxysteroid dehydrogenase type 1.
**MRs**: Mineralocorticoid receptors.
**NAD**: Nicotinamide adenine dinucleotide.

## References

[1] Werder E, et al., *Unusual steroid excretion in a child with low renin hypertension*. 1974. 6: p. 385–389.

[2] New MI (1977). Evidence for an unidentified steroid in a child with apparent mineralocorticoid hypertension. J Clin Endocrinol Metab.

[3] Nikkila H (1993). Defects in the HSD11 gene encoding 11 beta-hydroxysteroid dehydrogenase are not found in patients with apparent mineralocorticoid excess or 11-oxoreductase deficiency. J Clin Endocrinol Metab.

[4] Wilson RC (1995). A mutation in the HSD11B2 gene in a family with apparent mineralocorticoid excess. J Clin Endocrinol Metab.

[5] Mune T (1995). Human hypertension caused by mutations in the kidney isozyme of 11 beta-hydroxysteroid dehydrogenase. Nat Genet.

[6] Ulick S (1979). A syndrome of apparent mineralocorticoid excess associated with defects in the peripheral metabolism of cortisol. J Clin Endocrinol Metab.

[7] Nunez BS (1999). Mutants of 11beta-hydroxysteroid dehydrogenase (11-HSD2) with partial activity: improved correlations between genotype and biochemical phenotype in apparent mineralocorticoid excess. Hypertension.

[8] Dave-Sharma S (1998). Examination of genotype and phenotype relationships in 14 patients with apparent mineralocorticoid excess. J Clin Endocrinol Metab.

[9] Quinkler M (2004). Molecular basis for the apparent mineralocorticoid excess syndrome in the Oman population. Mol Cell Endocrinol.

[10] Ardhanari S (2015). Mineralocorticoid and apparent mineralocorticoid syndromes of secondary hypertension. Adv Chronic Kidney Dis.

[11] Cain DW, Cidlowski JA (2017). Immune regulation by glucocorticoids. Nat Rev Immunol.

[12] Liu B, et al., *The Glucocorticoid Receptor in Cardiovascular Health and Disease*. Cells, 2019. 8(10). 10.3390/cells8101227 PMC6829609 31601045

[13] Albiston AL (1994). Cloning and tissue distribution of the human 11 beta-hydroxysteroid dehydrogenase type 2 enzyme. Mol Cell Endocrinol.

[14] White PC, Mune T, Agarwal AK (1997). 11 beta-Hydroxysteroid dehydrogenase and the syndrome of apparent mineralocorticoid excess. Endocr Rev.

[15] Speiser PW (1993). Investigation of the mechanism of hypertension in apparent mineralocorticoid excess. Metabolism.

[16] Nimkarn S (2011). Apparent mineralocorticoid excess - update. Adv Exp Med Biol.

[17] Gomez-Sanchez EP, Gomez-Sanchez CE, *11beta-hydroxysteroid dehydrogenases: A growing multi-tasking family* Mol Cell Endocrinol, 2021. **526**: p. 111210. 10.1016/j.mce.2021.111210 PMC8108011 33607268

[18] Shams M (1998). 11Beta-hydroxysteroid dehydrogenase type 2 in human pregnancy and reduced expression in intrauterine growth restriction. Hum Reprod.

[19] Gennari-Moser C (2011). Regulation of placental growth by aldosterone and cortisol. Endocrinology.

[20] Stewart PM (1996). Hypertension in the syndrome of apparent mineralocorticoid excess due to mutation of the 11 beta-hydroxysteroid dehydrogenase type 2 gene. Lancet.

[21] Kwon YJ (2020). A Review of the Pharmacological Efficacy and Safety of Licorice Root from Corroborative Clinical Trial Findings. J Med Food.

[22] Beck KR (2020). Molecular mechanisms of posaconazole- and itraconazole-induced pseudohyperaldosteronism and assessment of other systemically used azole antifungals. J Steroid Biochem Mol Biol.

[23] Apostolakos JM, Caines LC (2016). Apparent Mineralocorticoid Excess Syndrome: A Case of Resistant Hypertension From Licorice Tea Consumption. J Clin Hypertens (Greenwich).

[24] Zahraldin K (2015). Two Qatari siblings with cystic fibrosis and apparent mineralocorticoid excess. Ann Thorac Med.

[25] Pizzolo F (2015). Apparent Mineralocorticoid Excess by a Novel Mutation and Epigenetic Modulation byHSD11B2Promoter Methylation. J Clin Endocrinol Metabolism.

[26] New MI (2005). Monogenic low renin hypertension. Trends Endocrinol Metab.

[27] Wilson RC (1995). Several homozygous mutations in the gene for 11 beta-hydroxysteroid dehydrogenase type 2 in patients with apparent mineralocorticoid excess. J Clin Endocrinol Metab.

[28] Yau M (2017). Clinical, genetic, and structural basis of apparent mineralocorticoid excess due to 11beta-hydroxysteroid dehydrogenase type 2 deficiency. Proc Natl Acad Sci U S A.

[29] New MI, Wilson RC (1999). Steroid disorders in children: congenital adrenal hyperplasia and apparent mineralocorticoid excess. Proc Natl Acad Sci U S A.

[30] Hammer F, Stewart PM (2006). Cortisol metabolism in hypertension. Best Pract Res Clin Endocrinol Metab.

[31] Palermo M, Quinkler M, Stewart PM (2004). Apparent mineralocorticoid excess syndrome: an overview. Arq Bras Endocrinol Metabol.

[32] Wilson RC (1998). A genetic defect resulting in mild low-renin hypertension. Proc Natl Acad Sci U S A.

[33] Moudgil A (2000). Nephrocalcinosis and renal cysts associated with apparent mineralocorticoid excess syndrome. Pediatr Nephrol.

[34] Abdulla MC, Narayan R, Ahamed S (2017). Renal Cysts and Nephrocalcinosis in 11 Beta-hydroxylase Deficiency. Indian J Nephrol.

[35] Carvajal CA (2018). Serum Cortisol and Cortisone as Potential Biomarkers of Partial 11beta-Hydroxysteroid Dehydrogenase Type 2 Deficiency. Am J Hypertens.

[36] Tapia-Castillo A (2021). Novel metabolomic profile of subjects with non-classic apparent mineralocorticoid excess. Sci Rep.

[37] Tapia-Castillo A (2019). Clinical, Biochemical, and Genetic Characteristics of “Nonclassic” Apparent Mineralocorticoid Excess Syndrome. J Clin Endocrinol Metab.

[38] Lavery GG (2003). Late-onset apparent mineralocorticoid excess caused by novel compound heterozygous mutations in the HSD11B2 gene. Hypertension.

[39] Morineau G (2006). Apparent mineralocorticoid excess: report of six new cases and extensive personal experience. J Am Soc Nephrol.

[40] Carvajal CA (2003). Two homozygous mutations in the 11 beta-hydroxysteroid dehydrogenase type 2 gene in a case of apparent mineralocorticoid excess. J Clin Endocrinol Metab.

[41] Bailey MA (2011). Hsd11b2 haploinsufficiency in mice causes salt sensitivity of blood pressure. Hypertension.

[42] Ueda K (2017). Renal Dysfunction Induced by Kidney-Specific Gene Deletion of Hsd11b2 as a Primary Cause of Salt-Dependent Hypertension. Hypertension.

[43] Tapia-Castillo A (2017). Hypertensive Patients That Respond to Aldosterone Antagonists May Have a Nonclassical 11beta-HSD2 Deficiency. Am J Hypertens.

[44] Parvez Y, Sayed OE (2013). Apparent mineralocorticoid excess (AME) syndrome. Indian Pediatr.

[45] Mantero F (1996). Apparent mineralocorticoid excess: type I and type II. Steroids.

[46] Palermo M, Cossu M, Shackleton CH (1998). Cure of apparent mineralocorticoid excess by kidney transplantation. N Engl J Med.

[47] Razzaghy-Azar M (2017). Apparent mineralocorticoid excess and the long term treatment of genetic hypertension. J Steroid Biochem Mol Biol.

[48] Agarwal AK (1995). Analysis of the human gene encoding the kidney isozyme of 11 beta-hydroxysteroid dehydrogenase. J Steroid Biochem Mol Biol.

[49] Kitanaka S (1997). A new compound heterozygous mutation in the 11 beta-hydroxysteroid dehydrogenase type 2 gene in a case of apparent mineralocorticoid excess. J Clin Endocrinol Metab.

[50] Lin-Su K (2004). In vitro expression studies of a novel mutation delta299 in a patient affected with apparent mineralocorticoid excess. J Clin Endocrinol Metab.

[51] Manning JR (2010). In silico structure-function analysis of pathological variation in the HSD11B2 gene sequence. Physiol Genomics.

[52] Atanasov AG (2007). Impaired protein stability of 11beta-hydroxysteroid dehydrogenase type 2: a novel mechanism of apparent mineralocorticoid excess. J Am Soc Nephrol.

[53] Mune T, White PC (1996). Apparent mineralocorticoid excess: genotype is correlated with biochemical phenotype. Hypertension.

[54] Wang Y (2017). Apparent mineralocorticoid excess caused by a novel mutation in 11beta-hydroxysteroid dehydrogenase type 2 gene. J Hypertens.

[55] Odermatt A (2001). A mutation in the cofactor-binding domain of 11beta-hydroxysteroid dehydrogenase type 2 associated with mineralocorticoid hypertension. J Clin Endocrinol Metab.

[56] Yau M (2016). A novel mutation in HSD11B2 causes apparent mineralocorticoid excess in an Omani kindred. Ann N Y Acad Sci.

[57] Obeyesekere VR (1997). Truncation of the N- and C-terminal regions of the human 11beta-hydroxysteroid dehydrogenase type 2 enzyme and effects on solubility and bidirectional enzyme activity. Mol Cell Endocrinol.

[58] Gomez-Sanchez EP (2001). The 11beta hydroxysteroid dehydrogenase 2 exists as an inactive dimer. Steroids.

[59] Woods C, Tomlinson JW (2015). The Dehydrogenase Hypothesis. Adv Exp Med Biol.

[60] Roland BL, Li KX, Funder JW (1995). Hybridization histochemical localization of 11 beta-hydroxysteroid dehydrogenase type 2 in rat brain. Endocrinology.

[61] Robson AC (1998). 11 Beta-hydroxysteroid dehydrogenase type 2 in the postnatal and adult rat brain. Brain Res Mol Brain Res.

[62] Ingram MC (1996). Sodium status, corticosteroid metabolism and blood pressure in normal human subjects and in a patient with abnormal salt appetite. Clin Exp Pharmacol Physiol.

[63] Evans LC (2016). Conditional Deletion of Hsd11b2 in the Brain Causes Salt Appetite and Hypertension. Circulation.

[64] Friso S (2008). Epigenetic control of 11 beta-hydroxysteroid dehydrogenase 2 gene promoter is related to human hypertension. Atherosclerosis.

[65] Alikhani-Koopaei R (2004). Epigenetic regulation of 11β-hydroxysteroid dehydrogenase type 2 expression. J Clin Invest.

[66] Marsit CJ (2012). Placental 11-beta hydroxysteroid dehydrogenase methylation is associated with newborn growth and a measure of neurobehavioral outcome. PLoS ONE.

[67] Majchrzak-Celinska A, et al., *HSD11B2, RUNX3, and LINE-1 Methylation in Placental DNA of Hypertensive Disorders of Pregnancy Patients* Reprod Sci, 2017. **24**(11): p. 1520–1531. 10.1177/1933719117692043 29017438

[68] Tapia-Castillo A (2019). Downregulation of exosomal miR-192-5p and miR-204-5p in subjects with nonclassic apparent mineralocorticoid excess. J Transl Med.

[69] De Santis D (2021). Detection of Urinary Exosomal HSD11B2 mRNA Expression: A Useful Novel Tool for the Diagnostic Approach of Dysfunctional 11beta-HSD2-Related Hypertension. Front Endocrinol (Lausanne).

[70] Alikhani-Koupaei R (2007). Identification of polymorphisms in the human 11beta-hydroxysteroid dehydrogenase type 2 gene promoter: functional characterization and relevance for salt sensitivity. Faseb j.

[71] Cai J (2017). Exposure to particulate air pollution during early pregnancy is associated with placental DNA methylation. Sci Total Environ.

[72] Raftopoulos L (2015). Epigenetics, the missing link in hypertension. Life Sci.

[73] Campino C (2013). Age-related changes in 11beta-hydroxysteroid dehydrogenase type 2 activity in normotensive subjects. Am J Hypertens.

[74] Najafi M (2019). Mimicry and well known genetic friends: molecular diagnosis in an Iranian cohort of suspected Bartter syndrome and proposition of an algorithm for clinical differential diagnosis. Orphanet J Rare Dis.

[75] Bouligand J (2010). Familial glucocorticoid receptor haploinsufficiency by non-sense mediated mRNA decay, adrenal hyperplasia and apparent mineralocorticoid excess. PLoS ONE.

[76] Narayanan R, et al., *Case Report: Application of whole exome sequencing for accurate diagnosis of rare syndromes of mineralocorticoid excess*. F1000Res, 2016. 5: p. 1592. 10.12688/f1000research.8779.1 PMC5635450 29067160

[77] Fan P (2020). Apparent mineralocorticoid excess caused by novel compound heterozygous mutations in HSD11B2 and characterized by early-onset hypertension and hypokalemia. Endocrine.

[78] Wilson RC (1995). Several homozygous mutations in the gene for 11 beta-hydroxysteroid dehydrogenase type 2 in patients with apparent mineralocorticoid excess. J Clin Endocrinol Metabolism.

[79] Al-Harbi T, Al-Shaikh A (2012). Apparent mineralocorticoid excess syndrome: report of one family with three affected children. J Pediatr Endocrinol Metab.

[80] Gulhan B, et al. Apparent mineralocorticoid excess: A diagnosis beyond classical causes of severe hypertension in a child. Blood Press Monit; 2022. 10.1097/MBP.0000000000000583 35044984

[81] Shackleton CH (1985). Congenital 11 beta-hydroxysteroid dehydrogenase deficiency associated with juvenile hypertension: corticosteroid metabolite profiles of four patients and their families. Clin Endocrinol (Oxf).

[82] DiMartino-Nardi J (1987). New findings in apparent mineralocorticoid excess. Clin Endocrinol (Oxf).

[83] Monder C (1986). The syndrome of apparent mineralocorticoid excess: its association with 11 beta-dehydrogenase and 5 beta-reductase deficiency and some consequences for corticosteroid metabolism. J Clin Endocrinol Metab.

[84] Coeli FB (2008). Apparent mineralocorticoid excess syndrome in a Brazilian boy caused by the homozygous missense mutation p.R186C in the HSD11B2 gene. Arq Bras Endocrinol Metabol.

[85] Milford DV, Shackleton CH, Stewart PM (1995). Mineralocorticoid hypertension and congenital deficiency of 11 beta-hydroxysteroid dehydrogenase in a family with the syndrome of ‘apparent’ mineralocorticoid excess. Clin Endocrinol (Oxf).

[86] Knops NB (2011). Apparent mineralocorticoid excess: time of manifestation and complications despite treatment. Pediatrics.

[87] Fiselier TJ (1982). Low-renin, low-aldosterone hypertension and abnormal cortisol metabolism in a 19-month-old child. Horm Res.

[88] Li A (1997). Apparent mineralocorticoid excess in a Brazilian kindred: hypertension in the heterozygote state. J Hypertens.

[89] Batista MC (1986). Spironolactone-reversible rickets associated with 11 beta-hydroxysteroid dehydrogenase deficiency syndrome. J Pediatr.

[90] Kitanaka S, Tanae A, Hibi I (1996). Apparent mineralocorticoid excess due to 11 beta-hydroxysteroid dehydrogenase deficiency: a possible cause of intrauterine growth retardation. Clin Endocrinol (Oxf).

[91] Li A (1998). Molecular basis for hypertension in the “type II variant” of apparent mineralocorticoid excess. Am J Hum Genet.

[92] Stewart PM (1988). Syndrome of apparent mineralocorticoid excess. A defect in the cortisol-cortisone shuttle. J Clin Invest.

[93] Bockenhauer D (2010). Secondary nephrogenic diabetes insipidus as a complication of inherited renal diseases. Nephron Physiol.

[94] Leventoglu E, et al. Late-onset hypertension in a child with growth retardation: Answers. Pediatr Nephrol; 2022. 10.1007/s00467-022-05510-8 35288793

[95] Alzahrani AS, et al., *Apparent Mineralocorticoid Excess Caused by a Novel Mutation in 11-beta Hydroxysteroid Dehydrogenase Type 2 Enzyme: Its Genetics and Response to Therapy*. Endocr Pract, 2014. 20(9): p. e151-6. 10.4158/EP14094.CR 24936560

[96] Adamidis A (2019). Apparent Mineralocorticoid Excess in the Pediatric Population: Report of a Novel Pathogenic Variant of the 11beta-HSD2 Gene and Systematic Review of the Literature. Pediatr Endocrinol Rev.

[97] Bertulli C, et al., *A Rare Cause of Chronic Hypokalemia with Metabolic Alkalosis: Case Report and Differential Diagnosis*. Children (Basel), 2020. 7(11). 10.3390/children7110212 PMC7694404 33167351

[98] Yadav M, et al., *Impaired Distal Tubular Acidification, Renal Cysts and Nephrocalcinosis in Monogenic Hypertension*. Indian J Pediatr, 2020. 10.1007/s12098-020-03516-4 33236328

